# Vector textures derived from higher order derivative domains for classification of colorectal polyps

**DOI:** 10.1186/s42492-022-00108-1

**Published:** 2022-06-14

**Authors:** Weiguo Cao, Marc J. Pomeroy, Zhengrong Liang, Almas F. Abbasi, Perry J. Pickhardt, Hongbing Lu

**Affiliations:** 1 Department of Radiology, State University of New York at Stony Brook, Stony Brook, NY 11794 USA; 2 Department of Biomedical Engineering, State University of New York at Stony Brook, Stony Brook, NY 11794 USA; 3 Department of Radiology, University of Wisconsin Medical School, Madison, WI 53705 USA; 4 Department of Biomedical Engineering, the Fourth Medical University, Xi’an, 710032 Shaanxi China

**Keywords:** Machine learning, Gradient, Hessian matrix, Haralick feature, Random forest, Image texture

## Abstract

Textures have become widely adopted as an essential tool for lesion detection and classification through analysis of the lesion heterogeneities. In this study, higher order derivative images are being employed to combat the challenge of the poor contrast across similar tissue types among certain imaging modalities. To make good use of the derivative information, a novel concept of vector texture is firstly introduced to construct and extract several types of polyp descriptors. Two widely used differential operators, i.e., the gradient operator and Hessian operator, are utilized to generate the first and second order derivative images. These derivative volumetric images are used to produce two angle-based and two vector-based (including both angle and magnitude) textures. Next, a vector-based co-occurrence matrix is proposed to extract texture features which are fed to a random forest classifier to perform polyp classifications. To evaluate the performance of our method, experiments are implemented over a private colorectal polyp dataset obtained from computed tomographic colonography. We compare our method with four existing state-of-the-art methods and find that our method can outperform those competing methods over 4%-13% evaluated by the area under the receiver operating characteristics curves.

## Introduction

Imaging tissue textures have become a widely researched topic in the field of medical diagnosis within recent years. As machine learning methods have grown through more powerful computers and computational algorithms, the research on tissue textures, particularly the lesion textures, has grown rapidly in the field because of the ever-expanding number of medical applications. One of the preliminary applications for texture analysis is lesion classification based on the heterogenous characteristics of the image contrast within and surrounding the lesion [1, 2]. Many studies have demonstrated that textures should be an important expression for the heterogeneities of medical images which describe some distinct morphological and phenotypic profiles and has become a critical measure in benign and malignant differentiability [3–8]. Using some texture measures computed from medical images, the tissue textures have demonstrated a powerful ability for computer-aided detection and diagnosis across a spectrum of diseases [9–11]. Among the most popular texture measures, some common examples include the gray-level co-occurrence matrix (GLCM) measure [12], gray-level run-length features [13], and the first order statistics features [14].

Research on tissue textures has shown the importance of the image intensity gradient within medical images [14–17]. By using the derivative operator on these medical images, the gradient information can be effectively encoded within the texture. The most notable examples of using the gradient information for textures are the histogram of oriented gradient (HOG) features [18] and the co-occurrence of local anisotropic gradient orientations (CoLIAGe) features [19]. The HOG features aim to bin the orientations of the gradients and use each histogram bin as an input feature for classification. The CoLIAGe features aim to compute the local entropy using the co-occurrence matrix (CM) of patches of voxels and the gradient angular information. The computed local entropy values are binned in a histogram, and then the histogram bins are used as input features for classification. These two methods have demonstrated a way of using the directional information in the gradient domain and coding the information into texture measures.

An alternative strategy was explored to investigate what additional information the higher order derivative images can provide beyond the original intensity image [20]. The idea is to magnify the original image contrast distribution at different orders for different texture patterns, aiming to encode as much information as possible about the lesion heterogeneity into quantitative texture measures. By using the first- and second-order derivatives to obtain the corresponding gradient and curvature images, exploratory studies [20, 21] provided an alternative way of encoding the higher order derivative image information into texture measures like GLCM.

While the above feature extraction methods have shown their potential in applications, they have their own limitations. For example, HOG method lacks any operation to extract information from the neighboring voxels that a CM-based method can provide. While the CoLIAGe method uses the CM, it limits itself only to the entropy values and considers only the local patches. The previous methods [20, 21] limit their use of GLCM to only the magnitude of the higher order images.

To address the limitations of the above methods, we further explore the use of higher order derivative information for polyp description and classification via computed tomographic colonography (CTC). Colorectal cancer (CRC) is one of the leading causes of cancer related deaths worldwide and accurate diagnosis remains a challenging task for radiologists [22]. While standard endoscopic colonoscopy is still the most popular screening method for CRC, CTC has grown to become a viable screening option to detect and diagnose both the precursor polyps and cancerous lesions. CTC is a non-invasive procedure which often makes it more palatable for patients, but unlike endoscopic colonoscopy, it cannot resect any concerning polyps. Accurate diagnosis through imaging textures of these polyp masses can better assist physicians to determine a plan of treatment while reducing costs for biopsy and pathology procedures [23], though computer-aided diagnosis of CTC polyps have been studied [21, 24, 25]. We focus on a dataset of polyp masses, or those polyps which have grown to be larger than 30 mm. These polyps require surgical intervention to remove, and the choice for how aggressively surgeons may cut into the colon for removal is determined by the malignancy of the lesion.

In this study, we look forward to expand upon the vector model introduced in ref. [26] to more deeply evaluate the image textures generated from the first- and second-order derivative information. Two differential operators, i.e., the gradient and Hessian operators, are employed to extract six local geometric measures, three of which are from the gradient operator and the other three are from the Hessian operator. The three local geometric measures of gradient operator are utilized to construct an angle-based and a vector (including both angle and magnitude)-based vector texture images (VTIs). Similarly, three local geometric measures of Hessian operator are utilized to construct an angle-based and a vector-based VTIs. From each VTI, a vector-based co-occurrence matrix (VCM) is proposed to compute a two-dimensional (2D) texture pattern along an angle through the VTI space, similar to the application of GLCM to the intensity images [12]. A series of 2D texture patterns is then obtained along different angles and called VCMs hereafter. From the computed series of 2D texture patterns or VCMs, several texture features can be derived and then fed to a classifier to perform the polyp classification.

The remainder of this study is organized as follows. Methods/experimental section describes the methods used to generate the new vector texture features (VTFs) and Results and discussion section presents the results obtained from all classification experiments using these new VTFs. Discussions and conclusions are drawn in last section.

## Methods/experimental

**Fig. 1:**
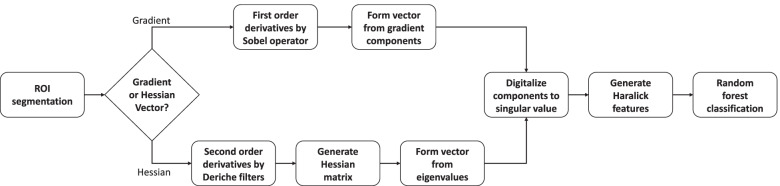
Flowchart of the proposed vector-texture method, showing the steps for either the gradient vector or Hessian vector approach

To overcome the challenges of limited soft tissue contrast from CT images, we first utilize the derivative operator to magnify the contrast. Then four VTIs are generated, two of which are from the gradient operator and the other two are from the Hessian operator. By applying the vector-based CM or VCM operator to each VTI, a series of VTFs are obtained and fed to a classifier. Figure 1 shows the flowchart of this work to outline the methods used to generate the derivative images, convert them into the associated vectors, and then input into a CM to form a set of vector-textures.

### Vector texture definition in gradient domain

**Fig. 2:**
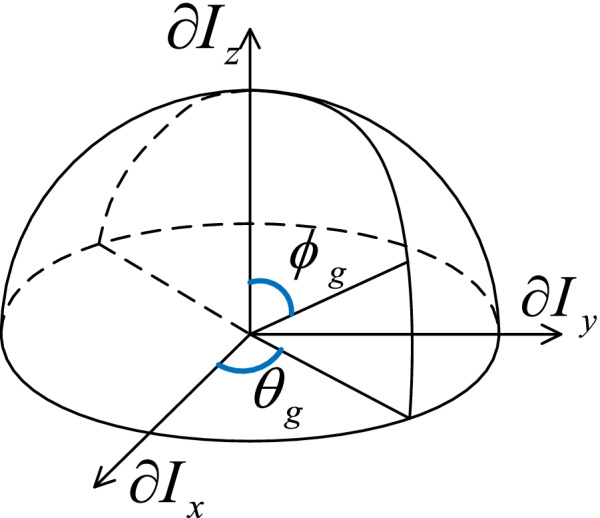
Diagram showing the azimuth and polar angles ${\theta }_{g}$ and ${\phi }_{g}$ respectively from a vector $\Delta I=\left(\partial {I}_{x}, \partial {I}_{y}, \partial {I}_{z}\right)$

Suppose a scalar function or intensity image $I=I(x,y,z)$ in three-dimensional (3D) space. Its gradient would be represented by the following vector function:

1 $$\Delta I=\left(\partial {I}_{x}, \partial {I}_{y}, \partial {I}_{z}\right)$$

where $\partial {I}_{x}, \partial {I}_{y},\mathrm{ and }\partial {I}_{z}$ are three partial differential parts. Since the intensity image is discrete and not continuous, we employ the well-established Sobel Operator kernels [27] to acquire the partial derivatives of the image. It is difficult to describe the object using the coordinates directly since the coordinate is a relative metric which could be changeable under different geometric transforms [28, 29]. Fortunately, a vector could be expressed by the geometric metrics of magnitude and direction. In 3D space, the direction of the vector has two components, the azimuth angle and polar angle, as shown in Fig. 2. To calculate the magnitude and direction of a gradient vector, a common method used is to translate the vector from Euclidean coordinates to spherical coordinates as follows:

2 $$\Delta I=\left(\partial {I}_{x}, \partial {I}_{y}, \partial {I}_{z}\right)= \left|\Delta I\right|\left(cos{\theta }_{g}cos{\phi }_{g},sin{\theta }_{g}cos{\phi }_{g},sin{\phi }_{g}\right)$$

where $\left|\Delta I\right|$ is the magnitude, ${\theta }_{g}$ is the azimuth angle and ${\phi }_{g}$ is the polar angle. Their definitions are given below:

3 $$\left|\Delta I\right|=\sqrt{{\left(\partial {I}_{x}\right)}^{2}+{\left(\partial {I}_{x}\right)}^{2}+{\left(\partial {I}_{z}\right)}^{2}}$$

4 $${\theta }_{g}=\left\{\begin{array}{cc}{\mathrm{cos}}^{-1}\left( \partial {I}_{x} \left/ \sqrt{{\left(\partial {I}_{x}\right)}^{2}+{\left(\partial {I}_{x}\right)}^{2}}\right.\right)& {\partial I}_{y}\ge 0\\ \pi +{\mathrm{cos}}^{-1}\left(\partial {I}_{x} \left/ \sqrt{{\left(\partial {I}_{x}\right)}^{2}+{\left(\partial {I}_{x}\right)}^{2}}\right.\right)& {\partial I}_{y}<0\end{array}\right.$$

5 $${\phi }_{g}={\mathrm{cos}}^{-1}\left(\partial {I}_{z}\left/ \sqrt{{\left(\partial {I}_{x}\right)}^{2}+{\left(\partial {I}_{x}\right)}^{2}+{\left(\partial {I}_{z}\right)}^{2}}\right.\right)$$

Unlike the CoLIAGe approach [19], where the azimuth and polar angles are used individually to extract texture features, we treat these two angles together as a description of gradient angle vector (GAV) of unit magnitude as:

6 $$GAV=\left({\theta }_{g},{\phi }_{g}\right)$$

where GAV only contains partial information of gradient assuming unit magnitude. In addition, we further employ the magnitude and direction to compose a total gradient vector (TGV), which preserves all gradient information:

7 $$TGV=\left(\left|\Delta I\right|,{\theta }_{g},{\phi }_{g}\right)$$

Our next step is to quantify the Formulas (6) and (7) to generate two VTIs. By their definitions, the range of the azimuth ${\theta }_{g}$ is $\left[0,\right.\left. 2\pi \right)$, and the range of the polar angle ${\phi }_{g}$ is in $\left[0,\right.\left. \pi \right)$. Their digitalization could be realized by:

8 $${\Theta }_{g}=\left\{\begin{array}{c}\left\lfloor \left({Q}_{g}^{a}\cdot {\theta }_{g}\right)\left/ 2\pi \right.\right\rfloor {\theta }_{g}\ne 2\pi \\ {Q}_{g}^{a}-1 {\theta }_{g}=2\pi \end{array}\right.$$

9 $${\Phi }_{g}=\left\{\begin{array}{c} \left\lfloor\left({Q}_{g}^{a}\cdot {\phi }_{g}\right) \left/ \pi \right.\right\rfloor {\phi }_{g}\ne \pi \\ {Q}_{g}^{p}-1 {\phi }_{g}=\pi \end{array}\right.$$

where ${Q}_{g}^{a}$ and ${Q}_{g}^{p}$ denote their gray levels, respectively, and $\lfloor{X}\rfloor$ represents the infimum of *X*.

**Fig. 3:**
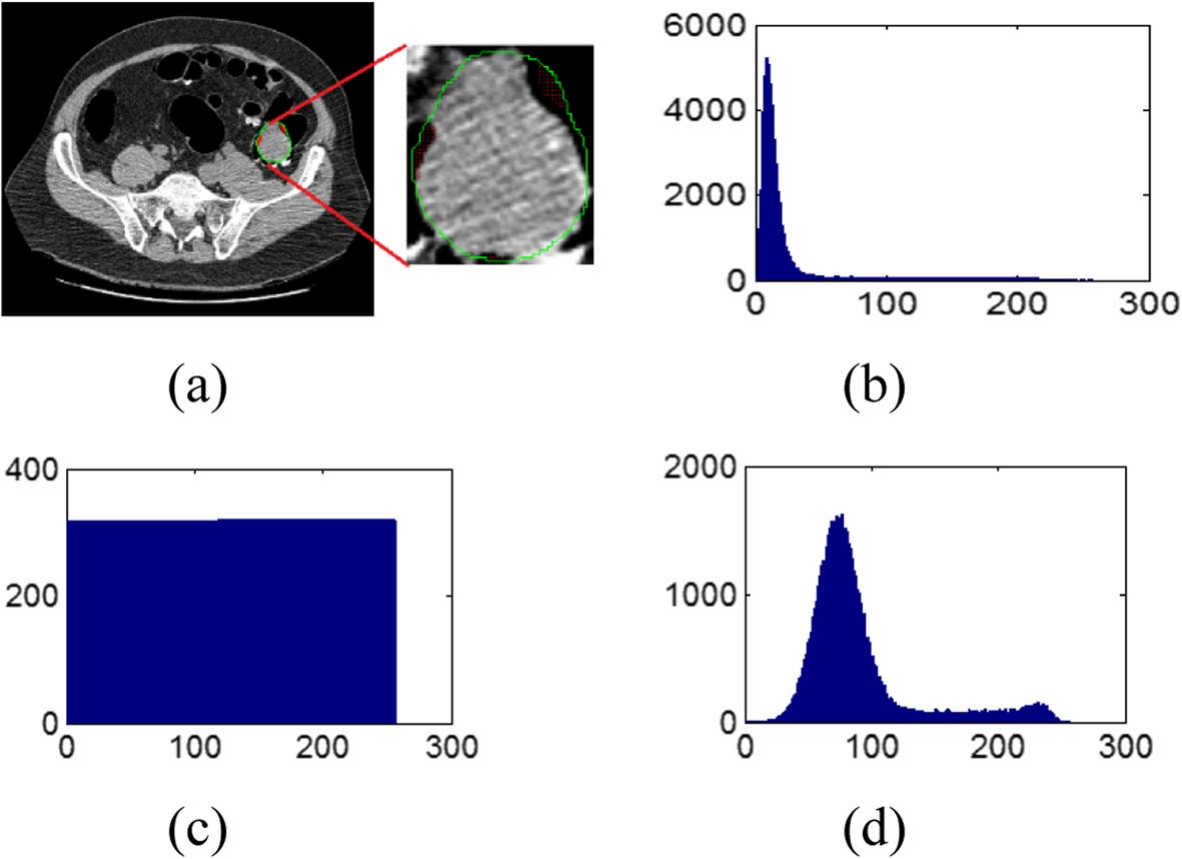
Gradient magnitude histogram with 256 Gy levels. (**a**): One slice from a 3D polyp volume; (**b**): Gradient magnitude under uniform gray scaling; (**c**): Gradient magnitude under non-uniform gray scaling; (**d**): Histogram after *t*-th root mapping with *t* = 3

The range of the gradient magnitude tends to be very wide, however most of that information is clustered within a narrow region. This can be seen in the distribution of magnitudes for our polyp dataset in Fig. 3b. Moreover, the CM is very sparse when computed from the unbalanced value distribution. A non-uniform sampling based on histogram equalization [30] can be a solution, which would generate very flat histogram as shown in Fig. 3c. This type of sampling methods treats every image component or region equally and ignores the different contributions from image component in different polyp subtypes. Hence, before quantification on the gradient image, we use a one-to-one mapping to change the pixel/voxel magnitude distribution by:

10 $${\Delta I}_{g}=\sqrt[t]{\left|\Delta I\right|}$$

where $\left|\Delta I\right|$ is the gradient magnitude of the original gradient image and *t* is an integer. Then a uniform gray scaling method is applied on ${\Delta I}_{g}$ by:

11 $${I}_{g}=\left\{\begin{array}{cc}\left\lfloor\left(\left({\Delta I}_{g}-{\Delta I}_{g}^{min}\right)\bullet {Q}_{g}^{m}\right)\left/ \left({\Delta I}_{g}^{max}-{\Delta I}_{g}^{min}\right)\right.\right\rfloor& {\Delta I}_{g}{\ne \Delta I}_{g}^{max}\\ {Q}_{g}^{m}-1& {\Delta I}_{g}{=\Delta I}_{g}^{max}\end{array}\right.$$

where ${\Delta I}_{g}$ was defined before as the re-mapped gradient magnitude, ${\Delta I}_{g}^{max}$ and ${\Delta I}_{g}^{min}$ are the maximum and minimum of ${\Delta I}_{g}$, and ${Q}_{g}^{m}$ is its maximum gray level number. Its histogram is shown in Fig. 3d.

After the quantification via Formulas (8, 9, 10 and11), we obtain two VTIs, corresponding to the definitions of GAV and TGV in the gradient image domain, as follows

12 $${T}_{1}=\left({\Theta }_{g} ,{\Phi }_{g}\right)$$

13 $${T}_{2}=\left({I}_{g},{\Theta }_{g} ,{\Phi }_{g}\right)$$

### Vector texture definition in Hessian domain

In mathematics, Hessian operator of a scalar function or intensity image $I=I(x,y,z)$ in 3D space could be defined by a 2^nd^ order derivative matrix as the follows:

14 $$H=\left[\begin{array}{ccc}{I}_{xx}& {I}_{xy}& {I}_{xz}\\ {I}_{xy}& {I}_{yy}& {I}_{yz}\\ {I}_{xz}& {I}_{yz}& {I}_{zz}\end{array}\right]$$

where *I*_*xx*_, *I*_*xy*_, *I*_*xz*_, *I*_*yy*_, *I*_*yz*_, and *I*_*zz*_ are the 2^nd^ order derivatives of $I(x,y,z)$. To compute these partial derivatives of the polyp images, we use the well-established Deriche filters [31] with parameter $\alpha =1$.

Due to the number of unique variables in the Hessian matrix we use matrix decomposition to reduce the dimensionality and extract the three eigenvalues ${\lambda }_{1}\ge {\lambda }_{2}\ge {\lambda }_{3}$. Since the Hessian matrix is a real symmetric matrix, this guarantees its eigenvalues and eigenvectors are all real values. We compose a 3D vector field, similar to the vector image construction in the gradient domain, as follows:

15 $$\mathrm{H}=\left({\lambda }_{1},{\lambda }_{2},{\lambda }_{3}\right)$$

**Fig. 4:**
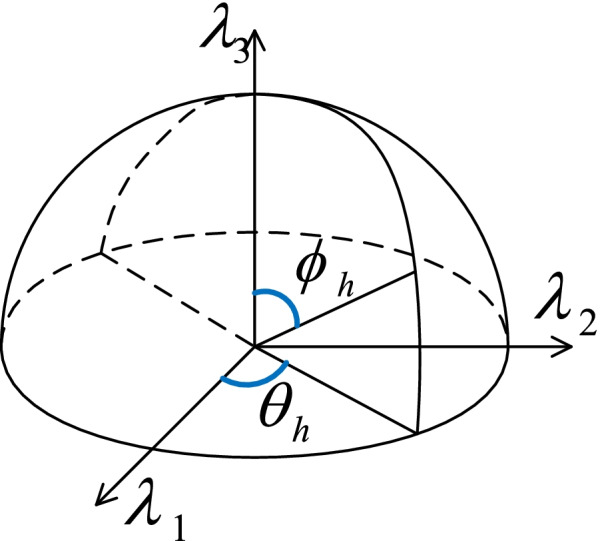
Diagram showing the azimuth and polar angles ${\theta }_{h}$ and ${\phi }_{h}$ respectively from a eigenvalue vector $\mathrm{H}=\left({\lambda }_{1},{\lambda }_{2},{\lambda }_{3}\right)$

Like the gradient, this vector could be expressed by spherical coordinates as below:

16 $$\mathrm{H}=\left({\lambda }_{1},{\lambda }_{2},{\lambda }_{3}\right)=|\uplambda |\left(cos{\theta }_{h}cos{\phi }_{h},sin{\theta }_{h}cos{\phi }_{h},sin{\phi }_{h}\right)$$

where |$\uplambda$| is the magnitude, ${\theta }_{h}$ and ${\phi }_{h}$ are two angles representing the direction as shown in Fig. 4. They are defined as follows:

17 $$|\uplambda |=\sqrt{{\left({\lambda }_{1}\right)}^{2}+{\left({\lambda }_{2}\right)}^{2}+{\left({\lambda }_{3}\right)}^{2}}$$

18 $${\theta }_{h}=\left\{\begin{array}{cc}{\mathrm{cos}}^{-1}\left({\lambda }_{1}\left/ \sqrt{{\left({\lambda }_{1}\right)}^{2}+{\left({\lambda }_{2}\right)}^{2}}\right.\right)& {\lambda }_{2}\ge 0\\ \pi +{\mathrm{cos}}^{-1}\left({\lambda }_{1}\left/ \sqrt{{\left({\lambda }_{1}\right)}^{2}+{\left({\lambda }_{2}\right)}^{2}}\right.\right)& {\lambda }_{2}<0\end{array}\right.$$

19 $${\phi }_{h}={\mathrm{cos}}^{-1}\left({\lambda }_{3}\left/ \sqrt{{\left({\lambda }_{1}\right)}^{2}+{\left({\lambda }_{2}\right)}^{2}+{\left({\lambda }_{3}\right)}^{2}}\right.\right)$$

Again, the Hessian angle vector (HAV) is defined as:

20 $$\mathrm{HAV}=\left({\theta }_{h},{\phi }_{h}\right)$$

The magnitude, azimuth angle and polar angle compose the total Hessian vector (THV) given by:

21 $$\mathrm{THV}=\left(\left|\uplambda \right|,{\theta }_{\mathrm{h}},{\phi }_{\mathrm{h}}\right)$$

Similar to the presentation in the gradient domain, we use the same method to perform the digitalization of ${\theta }_{h}$ and ${\phi }_{h}$ in the Hessian domain by:

22 $${\Theta }_{h}=\left\{\begin{array}{cc}\left\lfloor\left({Q}_{h}^{a}\bullet {\theta }_{h}\right)\left/ 2\pi \right.\right\rfloor & {\theta }_{h}\ne 2\pi \\ {Q}_{h}^{a}-1& {\theta }_{h}=2\pi \end{array}\right.$$

23 $${\Phi }_{h}=\left\{\begin{array}{cc}\left\lfloor\left({Q}_{h}^{p}\bullet {\phi }_{h}\right)\left/ \pi \right.\right\rfloor& {\phi }_{h}\ne \pi \\ {Q}_{h}^{p}-1& {\phi }_{h}=\pi \end{array}\right.$$

where ${Q}_{h}^{a}$ and ${Q}_{h}^{p}$ denote their gray levels or quantification numbers, respectively, and ⌊*X*⌋ was defined before as the infimum of *X*.

The gray scaling of Hessian magnitude is implemented by:

24 $${I}_{h}=\left\{\begin{array}{cc}\left\lfloor\left(\left({\Delta I}_{h}-{\Delta I}_{h}^{min}\right)\bullet {Q}_{h}^{m}\right) \left/ \left({\Delta I}_{h}^{max}-{\Delta I}_{h}^{min}\right) \right.\right\rfloor& {\Delta I}_{h}{\ne \Delta I}_{h}^{max}\\ {Q}_{h}^{m}-1& {\Delta I}_{h}{=\Delta I}_{h}^{max}\end{array}\right.$$

where ${\Delta I}_{h}=\sqrt[t]{|\uplambda |}$ is the re-mapped Hessian magnitude similar to Formula (10), ${\Delta I}_{h}^{max}$ and ${\Delta I}_{h}^{min}$ are the maximum and minimum of ${\Delta I}_{h}$, and ${Q}_{h}^{m}$ is the maximum gray level number.

Thus, another two VTIs, corresponding to the definitions of HAV and THV in the Hessian image domain, are obtained as follows:

25 $${T}_{3}=\left({\Theta }_{h} ,{\Phi }_{h}\right)$$

26 $${T}_{4}=\left({I}_{h},{\Theta }_{h} ,{\Phi }_{h}\right)$$

### Vector-based CM or VCM

Given above vector discretization, we present a new method based on the traditional GLCM called the vector-based CM, or VCM, and is expressed below as:

27 $${VCM}_{V_1,V_2}\left(d,\alpha,\beta\right)={\textstyle\sum_{s=1}^S}{\textstyle\sum_{r=1}^R}{\textstyle\sum_{c=1}^C}\left\{\begin{array}{cc}1&\begin{array}{c}T_i\left(r,c,s\right)=V_1\&\&\\T_i\left(\left(r,c,s\right)+d\ast(\alpha,\beta)\right)=V_2\end{array}\\0&otherwise\end{array}\right.$$

where ${T}_{i}$ represents the VTIs in 3D space, $i\in \{1, 2, 3, 4\}$, (R, C, S) is the volume size, ${V}_{1}$ and ${V}_{2}$ are a vector pair in ${T}_{i}$, and $d$ is the displacement between two voxels along the direction$\left(\alpha ,\beta \right)$, and $d*(\alpha ,\beta )=(d*cos\alpha cos\beta , d*sin\alpha cos\beta , d*sin\beta )$.

Given each of the four VTIs from Formulas (12), (13), (25), and (26), the Formula (27) generates a VCM along a direction in a similar way as the Haralick method [12] does. The Haralick method generates a GLCM along a direction from the original intensity image and our proposed method generates a VCM along a direction from the higher order VTIs. Because of this similarity, the Haralick method will be implemented as reference for comparison purpose.

A voxel within a 3D volume consists of 26 nearest neighbor voxels from which the VCM directions can be derived from. However, half of these directions produce redundant information as a transpose of their opposite direction. Therefore, only 13 directions are used for the VCM calculation and are denoted as: (0, 0, 1), (0, 1, 0), (1, 0, 0), (0, 1, 1), (1, 0, 1), (1, 1, 0), (-1, 1, 0), (0, 1, -1), (1, 0, -1), (1, 1, 1), (-1, 1, 1), (1, 1, -1) and (-1, 1, -1). Thus, using the VCM definition of Formula (27), we will compute 13 VCMs along 13 directions through a polyp volume [20]. By the use of the 28 texture measures from each direction [21], we will have total of 364 (= 13 × 28) texture measures per polyp. Since these 364 texture measures are derived from a 3D VTI of a polyp, they will be called VTFs hereafter. Classifying these VTFs and evaluating the classification outcomes are presented in the following section. The summary algorithm of generating these VTFs from for either the gradient vector or Hessian vector can be found below in Algorithm 1.


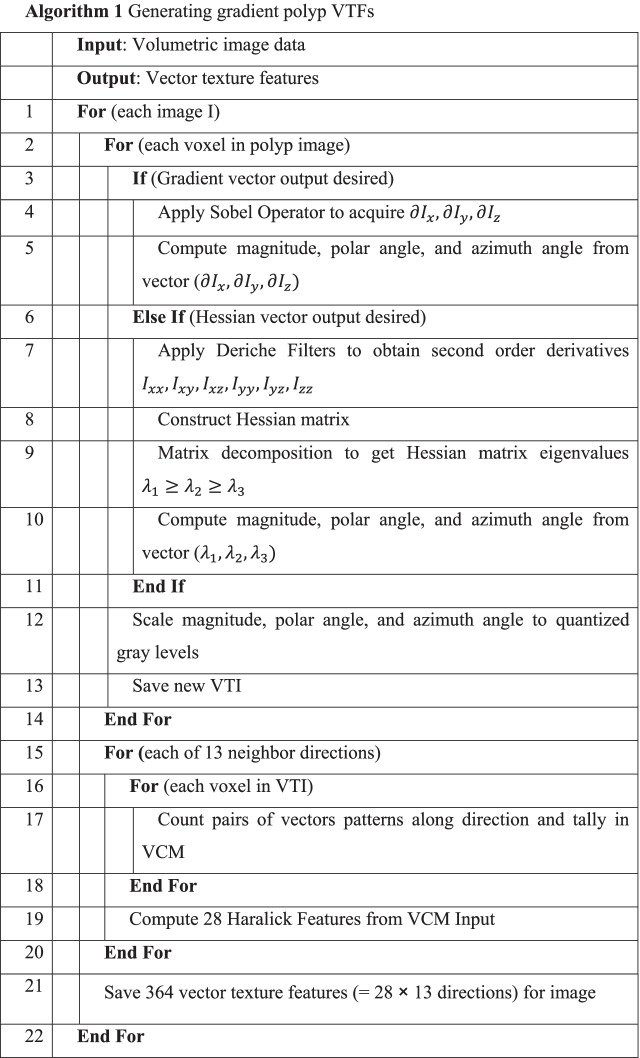


### Classification of the VTFs

Once the VTFs are computed for each polyp’s TVI, we use the R package ‘randomForest’ to perform classification using random forest (RF) [32, 33], as it has been shown to be effective in previous polyp classification experiments [34, 35]. Due to the limited data size of only 63 polyps, the RF method (and all machine learning algorithms) is susceptible to bias from the input data, or overfitting, depending on how it is divided into training and testing datasets. To reduce this overfitting effect, we utilize two different cross-validation methods to measure the robustness of the model. The first is a common twofold cross-validation method where we randomly divide the total dataset into equally sized testing and training sets, with the criteria that the ratio of benign and malignant lesions are maintained in each set. For a small dataset such as the one we are using, this method uses the least amount of training data and can therefore still lead to overfitting of the classification model. To alleviate this issue, we generate the random training and testing sets 100 times, and all results reported in Sect. 3 are the listed averages across all 100 randomly generated sets. For the second cross-validation scheme, we utilize a leave-one-out style approach where all except one polyp is used in the testing set, and the “left out” polyp is tested. This is repeated until all polyps have been left out of the training set and the final classification results summarize all iterations of the leave-one-out models. The results of the leave-one-out cross validation method are detailed in the Appendix.

To perform classification, we use the function ‘randomForest’ with the settings “ntreetry = 5000” and “doBest = True.” To perform the forward stepwise feature selection [36], we first do a preliminary classification using all available features and obtain the importance value for each feature by its GINI index [32]. The features are then sorted based on a decreasing GINI index value. The RF classification is then run with the 3 highest importance features, and repeated with iteratively increasing number of features added in importance order [21]. This procedure is repeated for each of the 100 randomized sets of testing and training data. The results are evaluated for each group, and the reported values show to average maximum area under the curve (AUC) of the receiver operating characteristic (ROC) curve. The results for the leave-one-out cross-validation method use this same feature selection procedure, only the distribution of training and testing sets are different.

Lastly, we note our choice of using RF classifier compared to other classifiers such as support vector machine (SVM) [37] or K-nearest neighbors [38]. Through internal experiments, we have generally found that we achieve better classification through RF over the other two methods. RFs have shown good classification in a variety of applications [39], and has been found that they tend to outperform SVM in instances of low resolution, such as for the dataset of polyp images used in this study [40]. Further, RF is probabilistic in its design, and is not under the same linear constraints required by SVM for its hyperplane segmentation. This work demonstrates the feasibility and efficacy of these proposed vector textures through RF classification, and comparisons across multiple classification modalities are subjects of future works.

### Results and discussion

In this section, we first describe the dataset and evaluate two parameters for gradient and Hessian magnitude to obtain some preliminary results for further experiments. Then polyp classification is performed using the VTFs extracted from the four VTIs. At the end, we compare our classification results with four existing methods.

### Polyp dataset

The data set used for these experiments consisted of 59 patients with a total number of 63 polyps found through CTC screening. These polyps were all at least 30 mm or larger that were scheduled for surgical removal later. When they were removed, the pathology reports were obtained to verify whether the polyps were indeed cancerous as an adenocarcinoma or were benign/pre-malignant as an adenomatous polyp. The breakdown of the dataset can be seen in Table 1. For classification, the dataset was divided into binary categories of malignant (adenocarcinoma) and pre-malignant (all others).

**Fig. 5:**
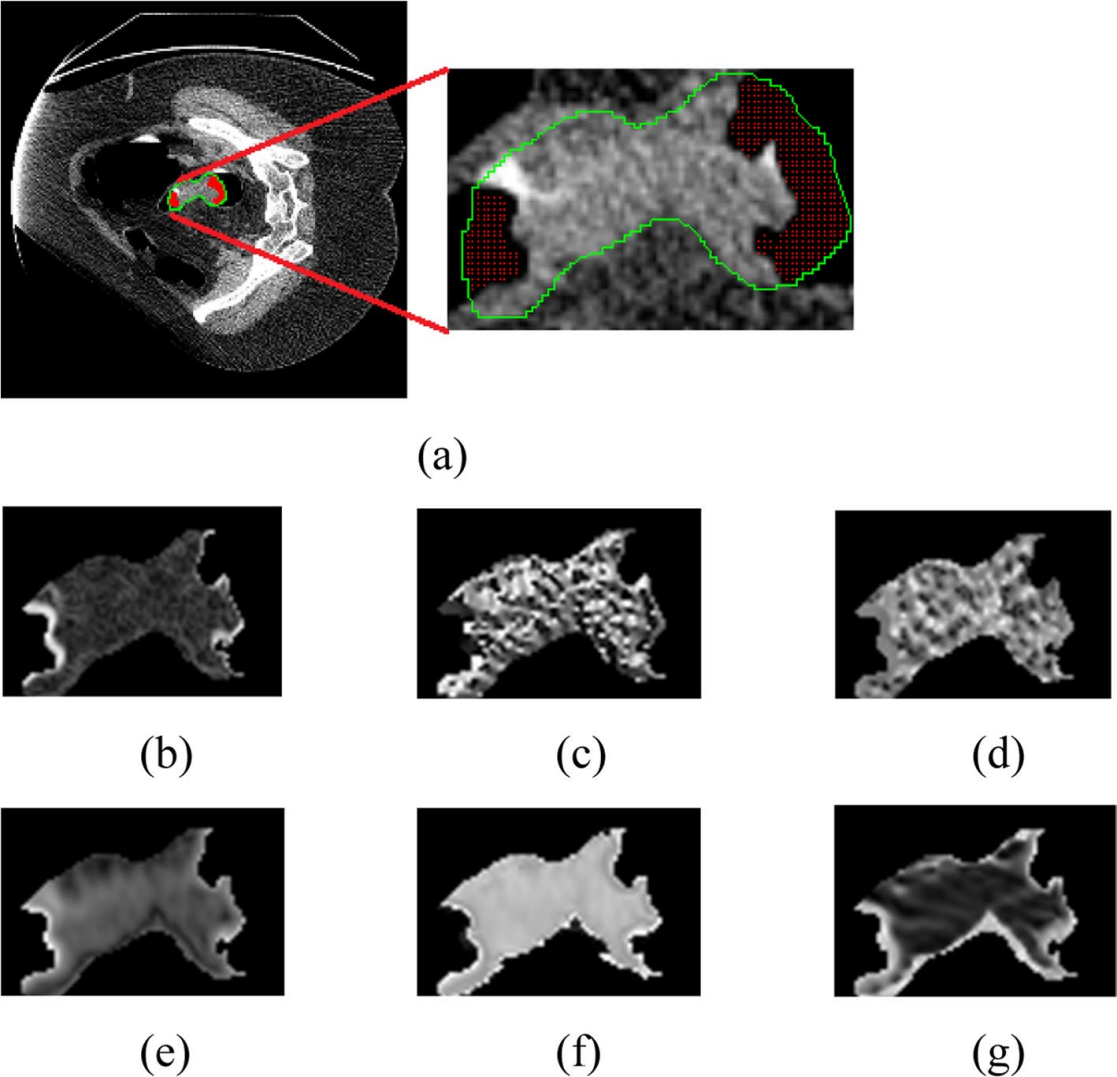
Sample slices of one polyp showing the different output variables with gray scale 256. (**a**): Original intensity; (**b**): Gradient magnitude; (**c**): Gradient azimuth angle; (**d**): Gradient polar angle; (**e**): Eigenvalue magnitude; (**f**): Eigenvalue azimuth angle; (**g**): Eigenvalue polar angle

The regions of interest (ROIs), used to compute the VTFs described below, were manually selected around the polyp region of the CTC image. For each polyp, a 3D volume was extracted, which was confirmed by radiologists to ensure accuracy for the manual procedure. We note that a thresholding preprocessing step was used to discard all voxels below -450 HU within these ROIs as being predominately air from the lumen of the colon. The information encoded in these voxels from partial volume effects is mostly air and therefore contributes more noise to the polyp features for classification. Since the first and second order derivatives apply kernels to the polyp images, the border voxels still use the intensity values provided outside of the ROI from the original CTC image. Examples of a polyp ROI slice are shown in Fig. 5 with each of the different image metrics used, i.e., the images in the original intensity domain, the gradient domain, and the Hessian (eigenvalue) domain. From the images, it is clear that each of the azimuth, polar, and magnitude images from the two derivative orders provide different types of information or different texture patterns. We hypothesize that given the same polyp descriptor or VCM in this study, the extracted VTFs from the gradient domain would be different from the Hessian domain, reflecting the polyp information at different orders.

While this dataset is small, we note the medical application to these polyp masses can still be quite significant. These polyp masses are large enough that removal will always be necessary, but the type of treatment may change based on the pathology. For example, accurate classification may help guide physicians to prevent unnecessary biopsy procedures which can help reduce medical costs and potential risks to the patient.

### Experimental results

#### Two parameters of magnitude images

Gradient magnitude and Hessian eigenvalue magnitude are two kinds of geometric measurements which represent the vector length. Since gradient and Hessian images both contain image edge information, they could enhance the contrast of the image to some extent, most notably at the boundaries. Therefore, their magnitude might vary in an interval with a wide range. However, most of the information is clustered in a very narrow interval as shown in Fig. 3. So, when we employ the *t*-th root mapping of Formula (10) to alleviate the issue, it is necessary to make sure how to set the value of *t* to obtain a top performance. To this goal, *t* is changed from 1 to 8 by the interval of 1 to calculate the polyp descriptors according to Formulas (5) and (12) while the gray level is kept 32. Table 2 lists the AUC scores of magnitudes of gradient and three Hessian eigenvalues. By consideration of their averages and their stabilities, we find *t* = 2 and *t* = 4 should be reasonable choices for gradient and Hessian magnitude images in the following experiments.

The second important parameter of magnitudes is its gray levels which determine the magnitude images and the VCM size. A larger gray level will demonstrate more details of metric images while smaller gray levels will over smooth the VCMs which will affect the discrimination of the texture features. Therefore, it is necessary to test the gray level’s influence in the polyp classification as shown in Table 3.

#### Outcomes of angle-based VCM

Both GAV and HAV are angle-based vector images which could be fed to VCM using Formula (27) to calculate the eHMs after digitalization. GAV represents the gradient angle vector image while HAV reflects the novel angle vector image derived from the Hessian eigenvalue vector. Both contain the two components of the azimuth and polar angles. In the angular vector digitalization, there are four parameters corresponding to the four angles, via ${Q}_{g}^{a}$,${Q}_{g}^{p}$,${Q}_{h}^{a}$ and ${Q}_{h}^{p}$ of Formulas (8), (9), (22) and (23). To avoid the matrix sparsity, we calculate the VCMs while ${Q}_{g}^{a}\in \left\{7, 8, 9, 10, 11\right\}$, ${Q}_{g}^{p}\in \left\{4, 5, 6\right\}$, and ${Q}_{h}^{a} and {Q}_{h}^{p}\in \left\{5, 6, 7, 8\right\}$. After classification via the RF classifier, their AUC scores are listed in Tables 4 and 5.

Table 4 illustrates that the GAV is very robust when the gray level varies from 28 to 66. The mean of AUC is about 0.94 while its standard deviation is almost less than 0.03. Comparatively, AUC scores of HAV in Table 5 show that its fluctuation of their averages is a little wider than GAV, which varies from 0.897 to 0.949. Meanwhile, most of the standard deviations are in the range of [0.03, 0.04]. From the best results of GAV and HAV, both could reach a similar classification level as high as 0.949.

#### Outcomes of vector (angle and magnitude)-based VCM

Dissimilar to GAV and HAV which only contain angles, TGV and THV consist of all information including both gradient and Hessian eigenvalues, i.e., the magnitudes and the azimuth and polar angles. Both vector images have six parameters in their quantization, i.e., ${Q}_{g}^{m}$, ${Q}_{g}^{a}$,${Q}_{g}^{p}$, ${Q}_{h}^{m}$,${Q}_{h}^{a}$ and ${Q}_{h}^{p}$. Like the angle-based VCM, we set some reasonable quantification levels for these parameters to generate non-sparse VCMs based on the best results of GAV and HAV. We hereby test TGV by changing ${Q}_{g}^{m}$ in the range {1, 2, 3, 4} while ${Q}_{g}^{a}$ keeps 10 and ${Q}_{g}^{p}$ varies in the range {4, 5} as shown in Table 6. Dissimilarly, we test ${Q}_{h}^{m}$ in {4, 5},${Q}_{h}^{a}$ in {3, 4} and ${Q}_{h}^{p}$ in {2, 3, 4, 5, 6, 7, 8} as shown in Table 7 where *t*-th power is equal to 4. To measure the classification performances, their AUC scores are obtained to show their effectiveness.

Table 6 demonstrates that TGV improves the average AUC scores and decreases their standard deviation compared with GAV’s classification results. That means all geometric components of gradient could provide some contribution to enhance the discrimination in polyp classification. Additionally, we find the similar trend for THV’s results in Table 7 which exceeds more than 1% by their best AUC scores.

### Comparison to other methods

For reference on how well our proposed method performs, we compare our results to some similar texture extraction methods and a state-of-the-art deep learning method as follows.

- Extended Haralick features (eHF) – this method extracts a set of 60 texture features from the GLCMs of the intensity image [21].
- HoG3D – this method counts the occurrences of gradient orientation in some cropped portions of the intensity image and generates some histograms which are joined to form gradient features [18].
- CoLIAGe – this model employs gradient angles to extract the entropy of every local patch to form global texture features by two joint histograms [19].
- VGG-16 – it is a widely cited deep learning method. Total of 20 salient slices were extracted from each polyp volume to feed to the VGG-16 pipeline for polyp classification [41].

Table 8 shows the results of comparison to the similar texture extraction methods listed above, where the same RF classifier was used, and the deep learning method. Our proposed method performed the best. We can see quite clearly that when comparing to the extended Haralick feature method, the *T*_*4*_ derived texture features outperformed the Haralick texture features with an AUC of 0.962 against 0.876. Comparing against other gradient angular features, our proposed angular texture features improved the performance substantially over the 3D HOG features (AUC = 0.804) and the CoLIAGe angular features (AUC = 0.923). The gain is also substantially higher over the VGG-16 outcome (AUC = 0.833). We note that deep learning methodologies such as VGG-16 have a much higher data requirement than conventional machine learning approaches. While we include the comparison to the VGG-16 model for comparison, we expect that the results of VGG-16 and other deep learning models such as those presented in refs. [42, 43] would be more comparable on a much larger dataset. Therefore, the eHF, HOG3D, and CoLIAGe models provide a more representative comparison and evaluation of the proposed vector-textures since they may be utilized under the same circumstances of fewer data entries.

Using a Wilcoxin ranked *t*-test, we obtained the quantitative measures for significant difference between the results of our proposed method and those of the reference methods as listed in Table 9. In all except one instance, we find that our results performed significantly better (*p* < 0.05) than the comparison methods.

## Conclusions

To enhance the image contrast of the original CTC polyp images, this study utilizes gradient operator and Hessian operator to generate the corresponding gradient image and Hessian image. The gradient image is represented by a vector field. The Hessian image is represented, according to the definition, by a matrix field. To avoid the difficulty of manipulating matrices, we take the three eigenvalues of the Hessian matrix at each image voxel as a vector and reduce the matrix field as a vector field similar to gradient field, thus all operations in the gradient domain are adapted to the Hessian domain. In addition, a novel concept of VTIs is proposed by the use of the vector geometric measures through the two vector fields in the corresponding gradient and Hessian domains, i.e., the GAV images (*T*_*1*_), the TGV images (*T*_*2*_), the HAV images (*T*_*3*_) and the THV image (*T*_*4*_). Moreover, another novel concept of vector-based CM or VCM is introduced to extract 2D texture patterns from these 2D/3D VTIs. These 2D texture patterns or VCMs can be viewed as the projection of the 2D/3D VTIs at different angles. From the projected VCMs, texture measures can be extracted as VTFs and classified by an existing classifier, such as RF as an example. Experimental outcomes demonstrated that the proposed VTF extraction method can outperform the state-of-the art feature extraction methods for polyp classification.

The novel textures introduced in this work are based on the CM. We had chosen to focus on the CM as previous studies demonstrated GLCM textures had typically outperformed those of related matrix-based textures such as those from gray-level run-length matrices. While we only focused on the co-occurrence-based matrices here, we believe these other matrices can be another avenue to explore these vector-textures and will be considered in our future works. We also note that these vector-textures place an emphasis on characterizing more microscopic properties of the lesions since they are based on the higher order derivative information. For classification with handcrafted features, it is important to introduce as much relevant information to the classifier while minimizing redundant information. Another topic for our future works will be looking into the integration of these vector-textures with other textures that emphasize more macroscopic lesion properties, such as the gray level size matrix. Appropriate feature selection methods will also be examined on how best to integrate these novel textures into the already existing library of handcrafted features.

**Table 1:** Characteristics of polyp data set

Class	Pathology	Total count	Male/female	Average size (mm)
Benign (0)	Serrated adenoma	3	2:1	34.3
	Tubular adenoma	2	2:0	35.0
	Tubulovillous adenoma	21	11:10	37.6
	Villous adenoma	5	4:1	55.0
Malignant (1)	Adenocarcinoma	32	12:20	43.9

**Table 2:** AUC scores of gradient magnitude and Hessian magnitude under different *t*-th root mappings

*t*-th root degree	Gradient magnitude	Hessian magnitude
1	0.882 ± 0.006	0.914 ± 0.006
2	0.898 ± 0.007	0.912 ± 0.004
3	0.899 ± 0.011	0.911 ± 0.005
4	0.893 ± 0.005	0.912 ± 0.004
5	0.892 ± 0.009	0.911 ± 0.005
6	0.891 ± 0.006	0.910 ± 0.005
7	0.886 ± 0.009	0.909 ± 0.005
8	0.885 ± 0.008	0.909 ± 0.005

**Table 3:** AUC scores of gradient magnitude and Hessian magnitude under different gray levels while the *t*-th root is set to be 2

Gray level	Gradient magnitude	Hessian magnitude
28	0.909 ± 0.046	0.929 ± 0.035
32	0.898 ± 0.007	0.912 ± 0.004
36	0.891 ± 0.041	0.924 ± 0.037
40	0.916 ± 0.038	0.923 ± 0.035
44	0.886 ± 0.042	0.921 ± 0.038
48	0.891 ± 0.043	0.940 ± 0.033
52	0.891 ± 0.043	0.926 ± 0.035
56	0.891 ± 0.039	0.938 ± 0.031
60	0.894 ± 0.043	0.930 ± 0.033
64	0.891 ± 0.042	0.938 ± 0.033
68	0.891 ± 0.040	0.922 ± 0.034

**Table 4:** AUC scores of GAV (or *T*_*1*_) with quantization between 28 and 66 and the *t*-th root is set to be 2

${{\varvec{Q}}}_{{\varvec{g}}}^{{\varvec{a}}}$	${{\varvec{Q}}}_{{\varvec{g}}}^{{\varvec{p}}}$	AUC (mean $\pm$ SD)
7	4	0.941 ± 0.025
7	5	0.930 ± 0.035
7	6	0.930 ± 0.027
8	4	0.943 ± 0.027
8	5	0.922 ± 0.030
8	6	0.934 ± 0.026
9	4	0.946 ± 0.027
9	5	0.944 ± 0.030
9	6	0.941 ± 0.033
10	4	0.946 ± 0.026
10	5	0.948 ± 0.029
10	6	0.946 ± 0.030
11	4	0.943 ± 0.028
11	5	0.943 ± 0.031
11	6	0.934 ± 0.032

**Table 5:** AUC values of HAV (or *T*_*3*_) with total quantization from 25 to 64 and the *t*-th root is set to be 2

${{\varvec{Q}}}_{{\varvec{h}}}^{{\varvec{a}}}$	${{\varvec{Q}}}_{{\varvec{h}}}^{{\varvec{p}}}$	AUC (mean $\pm$ SD)
5	5	0.912 ± 0.042
5	6	0.898 ± 0.039
5	7	0.915 ± 0.034
5	8	0.949 ± 0.026
6	5	0.920 ± 0.039
6	6	0.897 ± 0.038
6	7	0.927 ± 0.030
6	8	0.936 ± 0.033
7	5	0.933 ± 0.032
7	6	0.915 ± 0.037
7	7	0.926 ± 0.036
7	8	0.903 ± 0.037
8	5	0.921 ± 0.036
8	6	0.922 ± 0.040
8	7	0.937 ± 0.036
8	8	0.914 ± 0.039

**Table 6:** AUC scores of TGV (or *T*_*2*_) under different combinations of gradient magnitude, gradient azimuth and gradient polar angle where *t*-th root is set to be 2

${{\varvec{Q}}}_{{\varvec{g}}}^{{\varvec{m}}}$	${{\varvec{Q}}}_{{\varvec{g}}}^{{\varvec{a}}}$	${{\varvec{Q}}}_{{\varvec{g}}}^{{\varvec{p}}}$	AUC (mean $\pm$ SD)
1	10	5	0.948 ± 0.029
2	10	5	0.941 ± 0.033
3	10	5	0.934 ± 0.032
4	10	5	0.919 ± 0.035
1	10	4	0.946 ± 0.026
2	10	4	0.950 ± 0.025
3	10	4	0.949 ± 0.027

**Table 7:** AUC scores of THV (or *T*_*4*_) under different combinations of Hessian magnitude, Hessian azimuth and Hessian polar angle where the *t*-th root is set to be 4

${{\varvec{Q}}}_{{\varvec{h}}}^{{\varvec{m}}}$	${{\varvec{Q}}}_{{\varvec{h}}}^{{\varvec{a}}}$	${{\varvec{Q}}}_{{\varvec{h}}}^{{\varvec{p}}}$	AUC (mean ± SD)
4	3	6	0.913 ± 0.044
4	4	3	0.939 ± 0.034
4	4	4	0.951 ± 0.033
4	4	5	0.931 ± 0.036
4	4	8	0.954 ± 0.031
5	3	8	0.962 ± 0.027
5	4	2	0.912 ± 0.041

**Table 8:** AUC, accuracy, sensitivity, and specificity values from comparative methods and our proposed method where HF represents Haralick feature

Method	AUC	Accuracy	Sensitivity	Specificity
eHF	0.876	0.807	0.858	0.757
HOG3D	0.804	0.713	0.726	0.700
CoLIAGe	0.923	0.836	0.839	0.833
VGG-16	0.833	0.740	0.709	0.771
*T*_*1*_	0.948	0.868	0.883	0.853
*T*_*2*_	0.950	0.868	0.823	0.913
*T*_*3*_	0.949	0.863	0.847	0.879
*T*_*4*_	0.962	0.922	0.884	0.960

**Fig. 6:**
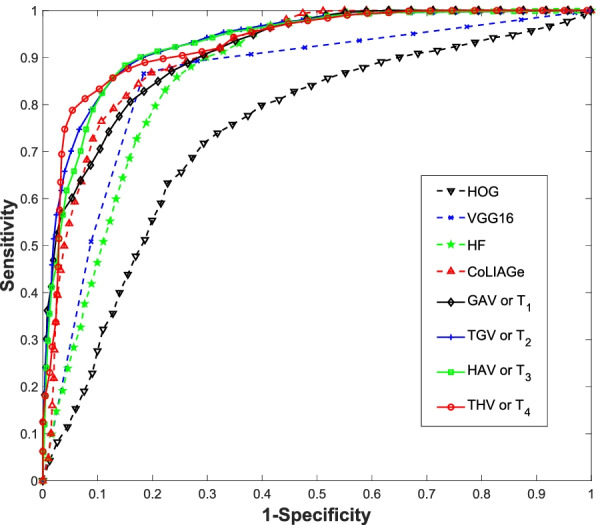
The ROC curves presented for each comparative method and our proposed method. For visual clarity, only the highest performing gradient vector and Hessian vector curves are shown where HF represents the Haralick features

Overall, the *T*_1_, *T*_2_, *T*_3_ and *T*_4_ derived texture features of our method achieved much higher AUC values than the four typical methods. The ROC curves of our four angular texture feature extraction methods are shown Fig. 6, which provides a visual assessment on their performances with comparison to other four references of HF, HOG, CoLIAGe and VGG-16

**Table 9:** *P*-values comparing proposed methods to comparison methods using Wilcoxin ranked sum test

Texture descriptor	CoLIAGe	HF	HOG3D	VGG-16
*T*_*1*_	< < 0.05	< < 0.05	< < 0.05	< < 0.05
*T*_*2*_	< < 0.05	< < 0.05	< < 0.05	< < 0.05
*T*_*3*_	< < 0.05	< < 0.05	< < 0.05	< < 0.05
*T*_*4*_	< < 0.05	< < 0.05	< < 0.05	< < 0.05

## Data Availability

The datasets used and analyzed during the current study are available from the corresponding author on reasonable request.

## Appendix

### Some experimental results using leave-one-out scheme

The results of using a leave-one-out cross validation method are presented below. For each model training instance in a leave-one-out method, only a single polyp is removed for the testing set at a time. The process is repeated until each polyp has been removed once, and the output classification probability from each polyp can be used to evaluate the overall performance. Since the leave-one-out method uses all but one polyp each instance, there is much more training data available to generate a model. This is especially important when the dataset used is already small with only 63 polyps. As can be seen in the tables below, the repeated experiments with a leave-one-out classifier generally perform better than the two-fold cross validation equivalent since there is more data in the training set each time.

The experiments for the results of Tables 4, 5, 6 and 7 of the two-fold cross validation method were repeated using the leave-one-out validation method and the outcomes are shown in Tables 10, 11, 12 and 13 respectively. We find for the GAV (or *T*_*1*_) model and the HAV (or *T*_*3*_) model, we can achieve AUC values of up to 0.960 and 0.975 respectively by changing the scaling parameters. We similarly find the TGV (or *T*_*2*_) and the THV (or *T*_*4*_) models can achieve AUC values up to 0.970 and 0.982 respectively. When comparing these values to those in Tables 4, 5, 6 and 7, we do find that the leave-one-out cross validation is able to obtain better overall classification performance by virtue of having more data available at each training instance.

**Table 10:** AUC scores of GAV (or *T*_*1*_) with quantization between 28 and 66 using a leave-one-out cross validation

${{\varvec{Q}}}_{{\varvec{g}}}^{{\varvec{a}}}$	${{\varvec{Q}}}_{{\varvec{g}}}^{{\varvec{p}}}$	AUC score
7	4	0.960
7	5	0.949
7	6	0.936
8	4	0.956
8	5	0.928
8	6	0.950
9	4	0.950
9	5	0.958
9	6	0.947
10	4	0.956
10	5	0.954
10	6	0.953
11	4	0.951
11	5	0.951
11	6	0.951

**Table 11:** AUC values of HAV (or *T*_*3*_) with total quantization from 25 to 64 using leave-one-out cross validation

${{\varvec{Q}}}_{{\varvec{h}}}^{{\varvec{a}}}$	${{\varvec{Q}}}_{{\varvec{h}}}^{{\varvec{p}}}$	AUC score
5	5	0.941
5	6	0.961
5	7	0.917
5	8	0.975
6	5	0.956
6	6	0.971
6	7	0.947
6	8	0.954
7	5	0.921
7	6	0.918
7	7	0.938
7	8	0.939
8	5	0.973
8	6	0.943
8	7	0.960
8	8	0.941

**Table 12:** AUC scores of TGV (or *T*_*2*_) under different combinations of gradient magnitude, gradient azimuth and gradient polar angle using leave-one-out cross validation

${{\varvec{Q}}}_{{\varvec{g}}}^{{\varvec{m}}}$	${{\varvec{Q}}}_{{\varvec{g}}}^{{\varvec{a}}}$	${{\varvec{Q}}}_{{\varvec{g}}}^{{\varvec{p}}}$	AUC score
1	10	5	0.954
2	10	5	0.948
3	10	5	0.921
4	10	5	0.924
1	10	4	0.956
2	10	4	0.970
3	10	4	0.953

**Table 13:** AUC scores of THV (or *T*_*4*_) under different combinations of Hessian magnitude, Hessian azimuth and Hessian polar angle using leave-one out cross validation

${Q}_{h}^{m}$	${Q}_{h}^{a}$	${Q}_{h}^{p}$	AUC score
4	3	6	0.922
4	4	3	0.966
4	4	4	0.982
4	4	5	0.957
4	4	8	0.961
5	3	8	0.968
5	4	2	0.936

## Abbreviations

HOG: Histogram of oriented gradient
CoLIAGe: Co-occurrence of local anisotropic gradient orientations
CM: Co-occurrence matrix
GLCM: Gray-level co-occurrence matrix
CRC: Colorectal cancer
CTC: Computed tomographic colonography
VTI: Vector texture image
VCM: Vector-based co-occurrence matrix
GAV: Gradient angle vector
TGV: Total gradient vector
HAV: Hessian angle vector
THV: Total Hessian vector
VTF: Vector texture feature
eHF: Extended Haralick features
2D: Two-dimensional
3D: Three-dimensional
AUC: Area under the curve
ROI: Region of interest
ROC: Receiver operating characteristic
SVM: Support vector machine
